# Neural Surrogate-Enhanced Metaheuristic Optimization for Distributed Quadrotor Swarm Control

**DOI:** 10.3390/s26113398

**Published:** 2026-05-27

**Authors:** Jinze Li, Zeling Wen, Zhaoke Ning

**Affiliations:** 1 School of Aeronautics and Astronautics, Sichuan University, Chengdu 610207, China; 2023141510085@stu.scu.edu.cn (J.L.); coollingwen@gmail.com (Z.W.); 2 Multi-Source Information Intelligent Fusion Key Laboratory of Sichuan Province, Chengdu 610207, China

**Keywords:** UAV swarms, multi-objective optimization, neural surrogate, distributed control, real-time decision making

## Abstract

Real-time cooperative control of quadrotor swarms in cluttered environments requires balancing formation maintenance, obstacle avoidance, inter-UAV safety, and per-step computational cost. This paper proposes a multilayer perceptron (MLP) surrogate for high-level objective-weight selection in a modified multi-objective pigeon-inspired optimization (modified MPIO) distributed controller. The proposed MLP surrogate learns the state-to-weight mapping of the online search and directly predicts the two-dimensional objective-weight vector, while the original flocking, gap-based obstacle-avoidance, and command generation rules are retained unchanged. The surrogate is trained from teacher-generated weight labels using randomized scenes, DAgger-based state aggregation, and risk-weighted supervision. On a fixed closed-loop benchmark, the proposed controller increases the true collision free rate from 48.00% to 86.89% and the safe success rate from 38.67% to 74.22% relative to modified MPIO, while reducing the mean per-step decision latency for the whole swarm from 8494.70 ms to 0.92 ms. The improvement is most pronounced in safety-related and runtime metrics, while the formation-related gain is comparatively modest. Ablation results show that the final benchmark performance is not explained by DAgger or risk weighting alone, and that the medium-sized surrogate provides the best safety-latency tradeoff among the tested network architectures. A qualitative AirSim case study further indicates that the same high-level surrogate controller can be executed in a higher-fidelity asynchronous multirotor simulator.

## 1. Introduction

Autonomous path planning for single UAVs has been studied extensively; the central problem is to generate safe and dynamically feasible trajectories from available map or sensor information while reaching mission objectives [1]. Compared with the single-UAV case, multi-UAV planning introduces additional coupling among agents: multiple vehicles must reach mission goals while maintaining coordination, inter-agent safety, and robustness under sensing and communication constraints [2,3,4,5]. Therefore, the resulting problem is a coupled multi-agent control problem in cluttered and time-varying environments rather than a single-route generation problem.

Representative structured coordination lines include flocking and consensus rules [6,7,8], velocity–obstacle methods [9,10], and safety certificate-based collision avoidance [11]. Optimization-based and heuristic or metaheuristic methods remain attractive for cooperative UAV control because they can accommodate nonconvex objectives, coupled constraints, and competing mission requirements without relying on a fully tractable analytical model [12,13,14,15]. Representative multi-objective UAV studies have shown that these methods can produce feasible cooperative behaviors in complex scenes [16,17]. Their main limitation for deployment is computational; when optimization must be executed online at every control step, the search cost grows quickly with swarm size, obstacle density, and constraint coupling, making strict real-time execution difficult [3,14].

Recent studies in adjacent domains involving dynamic control also reflect the broader use of learning and iterative optimization to improve closed-loop control under practical constraints, including model-free Q-learning-based fault-tolerant control for batch processes [18] and infinite-horizon iterative learning control under actuator failures [19]. Although these works address different plants and task structures from decentralized quadrotor swarm obstacle avoidance, they further motivate the general need to reduce online decision burden while preserving closed-loop control performance.

When repeated online optimization becomes the main bottleneck, learning-based methods become a practical way to reduce online computation. Related approximation routes include explicit MPC [20], learning-based MPC [21], and neural approximations of optimization-based controllers [22,23]. However, end-to-end replacement also weakens interpretability and explicit safety semantics in safety-critical multi-UAV control [21,24]. In closed-loop control, distribution shift is another key issue. Behavior-cloning policies can degrade when deployment states deviate from the training distribution. DAgger addresses this by aggregating expert labels on learner-visited states [25,26]. These considerations make full policy replacement less attractive in our setting, and instead point to a more limited surrogate design that preserves the existing control structure while replacing only the heaviest online decision module.

Therefore, this paper adopts a module-level neural-surrogate design. We retain the inherited distributed control structure and replace only the online modified MPIO search over a two-dimensional objective-weight vector. This weight vector regulates the tradeoff between flocking/formation maintenance and obstacle-avoidance behavior at each control step. Therefore, the neural surrogate does not output direct UAV control commands; it only predicts this intermediate decision variable, which is then used by the unchanged downstream control rules. Because the learned variable is low-dimensional and interpretable, it can be supervised by teacher-generated weight labels while preserving the surrounding control semantics. The surrogate is trained with randomized scenes, DAgger-style state aggregation, and risk-weighted supervision, then evaluated on a retained synchronous closed-loop benchmark. AirSim is used only as a qualitative case study for higher-fidelity asynchronous multirotor execution [26,27]. Our main contributions are as follows:

(1) We address the deployment-time bottleneck in an inherited modified MPIO swarm controller by replacing its two-dimensional online weight selection module with a lightweight neural surrogate, leaving the existing flocking and gap-based obstacle-avoidance rules unchanged.
(2) We train the surrogate with scene randomization, DAgger, and risk-weighted supervision, allowing it to better handle learner-visited states and place greater emphasis on safety-related samples during training.
(3) We evaluate the method on a fixed synchronous closed-loop benchmark as well as a qualitative AirSim case study. On the benchmark, the surrogate improves the true collision-free rate and safe success rate while greatly reducing whole-swarm per-step decision latency and eliminating step overruns under the current implementation. In AirSim, the same high-level controller remains executable in an asynchronous multirotor control loop, providing qualitative evidence of migration feasibility.
(4) We release the source code, final trained model, and merged evaluation results to support reproducibility and further comparison (https://github.com/cliche71/quadrotor-swarm-neural-surrogate.git, accessed on 17 May 2026).

## 2. System Modeling and Problem Formulation

### 2.1. UAV Model

At the platform level, each agent is a quadrotor Unmanned Aerial Vehicle (UAV). Because the eventual deployment target is a quadrotor swarm rather than an abstract point robot, we briefly state a standard six-degree-of-freedom (6-DOF) rigid-body model to define the vehicle variables and execution layer, following Sabatino’s thesis [28] and the survey by Zhang et al. [29]. Let $\left\{O_{W}\right\}$ denote the inertial frame and $\left\{O_{B}\right\}$ the body-fixed frame. The UAV position is described by $p_{W}$$={\left[x,y,z\right]}^{⊤}$ and its attitude is represented by the Euler angles (ϕ,θ,ψ), where (x,y,z) are the inertial coordinates of the center of mass and (ϕ,θ,ψ) are the roll, pitch, and yaw angles, respectively.

The world-frame translational kinematics are

$${\dot{p}}_{W}=v_{W},\;v_{W}={\left[\dot{x},\dot{y},\dot{z}\right]}^{⊤}.$$

The body-frame linear and angular velocities are

$$v_{B}={\left[u,v,w\right]}^{⊤},\;\omega_{B}={\left[p,q,r\right]}^{⊤}.$$

The mass is denoted by *m*, the inertia matrix by $J=\mathrm{diag}(J_{x},J_{y},J_{z})$, the total thrust along the body $z_{B}$-axis by *T*, and the body torques by $\tau_{B}$$\;={\left[\tau_{\phi },\tau_{\theta },\tau_{\psi }\right]}^{⊤}$. Using the standard Euler-angle attitude representation, the full 6-DOF quadrotor model can be written as follows [28,29]:

(1) $$\left\{\begin{array}{cc} \ddot{x} & =\frac{T}{m}\left(\cos\phi \sin\theta \cos\psi +\sin\phi \sin\psi \right),\\ \ddot{y} & =\frac{T}{m}\left(\cos\phi \sin\theta \sin\psi -\sin\phi \cos\psi \right),\\ \ddot{z} & =\frac{T}{m}\cos\phi \cos\theta -g,\\ \dot{\phi } & =p+q\sin\phi \tan\theta +r\cos\phi \tan\theta ,\\ \dot{\theta } & =q\cos\phi -r\sin\phi ,\\ \dot{\psi } & =q\frac{\sin\phi }{\cos\theta }+r\frac{\cos\phi }{\cos\theta },\\ \dot{p} & =\frac{1}{J_{x}}\;\left(\tau_{\phi }+\left(J_{y}-J_{z}\right)qr\right),\\ \dot{q} & =\frac{1}{J_{y}}\;\left(\tau_{\theta }+\left(J_{z}-J_{x}\right)pr\right),\\ \dot{r} & =\frac{1}{J_{z}}\;\left(\tau_{\psi }+\left(J_{x}-J_{y}\right)pq\right). \end{array}\right.$$

**Figure 1:**
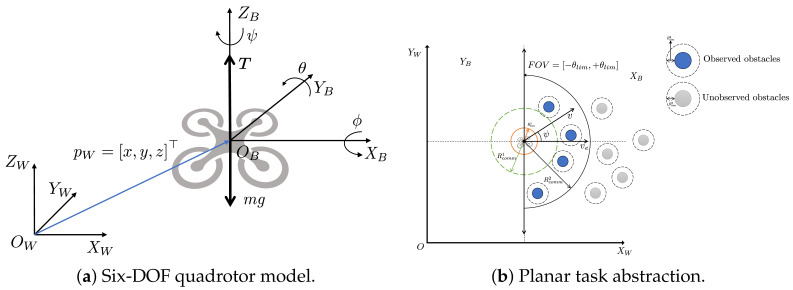
Quadrotor model and planar task abstraction.

However, the present contribution is not a new low-level attitude controller; rather, the optimization and learning developments of this paper operate at the high-level swarm-decision layer. For the planar task considered in this work, the horizontal motion of UAV *i* is described by

$$p_{i}={\left[x_{i},\;y_{i}\right]}^{⊤},$$

where $x_{i}$ and $y_{i}$ denote the planar coordinates. Under the adopted heading-angle definition, the planar kinematics are

$${\dot{p}}_{i}=\left[\begin{array}{c} {\dot{x}}_{i}\\ {\dot{y}}_{i} \end{array}\right]=v_{i}\left[\begin{array}{c} \cos\psi_{i}\\ \sin\psi_{i} \end{array}\right],$$

where $v_{i}=∥v_{i}∥$ is the planar speed and $\psi_{i}$ is the heading angle. Figure 1 illustrates the 6-DOF quadrotor coordinate frames and the planar task abstraction used in this work.

### 2.2. Swarm Flocking Objectives

We consider a swarm of *N* UAVs indexed by i∈{1,…,N}. The horizontal position and velocity of UAV *i* are denoted by $p_{i}={\left[x_{i},y_{i}\right]}^{⊤}\in R^{2}$ and $v_{i}={\left[v_{x,i},v_{y,i}\right]}^{⊤}\in R^{2}$, respectively. Each UAV interacts only with neighbors inside a limited communication radius $R_{\mathrm{comm}}$, leading to the neighbor set

(2) $$N_{i}≜\left\{\;j\neq i\;|\;∥p_{j}-p_{i}∥\leq R_{\mathrm{comm}}\right\}.$$

Following classical flocking models and distributed coordination principles [7,30], the present model adopts a local-rule view in which spacing regulation, velocity alignment, and short-range repulsion jointly shape the collective motion of the swarm. Under this view, flocking is not treated as a purely emergent effect, but as a structured high-level control prior written explicitly in terms of neighborhood interactions and local safety-related forces. In the current controller semantics, three horizontal control primitives are computed for UAV *i*: a spacing (cohesion–separation) term ${\dot{v}}_{i}^{\mathrm{space}}$, a velocity alignment term ${\dot{v}}_{i}^{\mathrm{align}}$, and a short-range collision repulsion term ${\dot{v}}_{i}^{\mathrm{coll}}$.

The weighted spacing contribution used in the flocking channel drives $d_{ij}$ towards a desired spacing radius $R_{\mathrm{des}}$:

(3) $${\dot{v}}_{i}^{\mathrm{space}}=w_{i1}K_{f}\;\;\sum_{j\in N_{i}}\left(1-{\left(\frac{R_{\mathrm{des}}}{d_{ij}}\right)}^{2}\right)\;\Delta p_{ij},$$

where $K_{f}$ is the spacing gain. When $d_{ij}>$$R_{\mathrm{des}}$, the coefficient in parentheses is positive and ${\dot{v}}_{i}^{\mathrm{space}}$ attracts UAV *i* towards its neighbors, whereas for $d_{ij}<$$R_{\mathrm{des}}$ it becomes negative and induces repulsion; $\Delta p_{ij}=p_{j}-p_{i}$ and $d_{ij}=∥\Delta p_{ij}∥$ denote the relative position and distance from agent *i* to *j*.

The weighted alignment contribution reduces velocity disagreement among neighbors and promotes locally coherent motion with alignment gain $K_{a}$:

(4) $${\dot{v}}_{i}^{\mathrm{align}}=w_{i1}K_{a}\;\;\sum_{j\in N_{i}}\left(v_{j}-v_{i}\right).$$

To prevent close-proximity contact under dense interactions, we further introduce a strong repulsive term when another UAV enters a safety radius $R_{\lim}^{1}$ around agent *i*:

(5) $${\dot{v}}_{i}^{\mathrm{coll}}=-K_{c}\;\;\;\sum_{j\in N_{i}:\;d_{ij}\leq R_{\lim}^{1}}{\left(\frac{1}{d_{ij}}-\frac{1}{R_{\lim}^{1}}\right)}^{2}\frac{\Delta p_{ij}}{d_{ij}},$$

where $K_{c}$ is the collision avoidance gain. This term grows rapidly as the inter-UAV distance decreases, and as such provides a hard local safety buffer.

The horizontal flocking contribution used later in the controller is then given by

(6) $${\dot{v}}_{i}^{\mathrm{flock}}={\dot{v}}_{i}^{\mathrm{space}}+{\dot{v}}_{i}^{\mathrm{align}}+{\dot{v}}_{i}^{\mathrm{coll}}.$$

### 2.3. Obstacles and Gap-Based Avoidance Model

We model obstacles on the flight plane at altitude $h_{e}$ as inflated disks with center $o_{k}={\left[x_{k},y_{k}\right]}^{⊤}$ and effective safety radius $R_{\lim,k}^{2}$, indexed by $k\in K_{\mathrm{obs}}$. For static cylindrical obstacles, each disk is given by the cylinder–plane intersection. For moving spherical obstacles, the disk center is updated at each control step from the obstacle state on that plane, with the local avoidance decision being recomputed from the refreshed obstacle positions. Accordingly, the planar obstacle region considered by the local avoidance module is

(7) $$O_{k}=\left\{p\in R^{2}\;|\;∥p-o_{k}∥\leq R_{\lim,k}^{2}\right\}.$$

**Figure 2:**
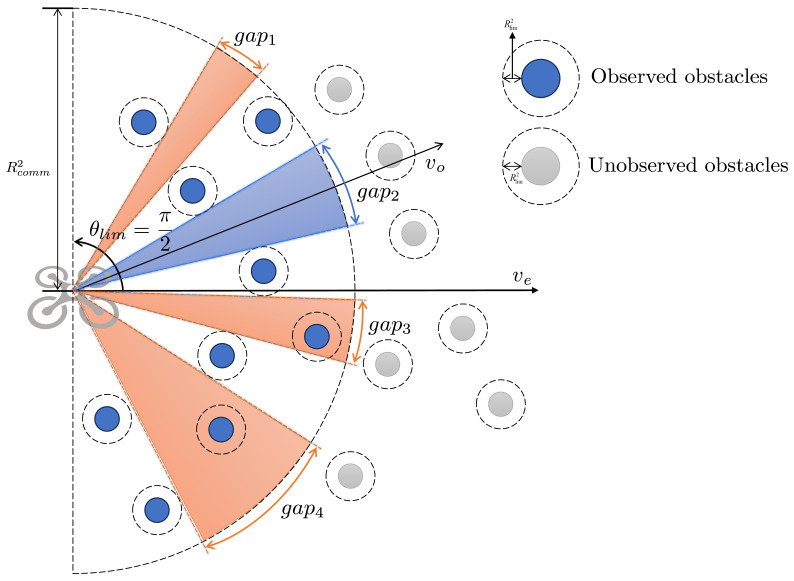
Local perception model for obstacles and gap-based avoidance.

The local perception geometry is illustrated in Figure 2.

Each UAV is equipped with a forward-looking depth sensor with sensing range $R_{\mathrm{sense}}$ and field of view $\left[-\Theta_{\mathrm{FOV}},\Theta_{\mathrm{FOV}}\right]$. Let $v_{e}={\left[v_{e,x},v_{e,y}\right]}^{⊤}$ denote the desired planar cruise velocity. The current local heading $\psi_{i}$ is taken from the current horizontal velocity direction; when the velocity is near zero, the UAV falls back on the direction of $v_{e}$.

In the local angular frame centered at $\psi_{i}$, each detected obstacle induces a blocked angular interval, and the complement of the union of all blocked intervals defines the free-gap set:

(8) $$G_{i}=\left[-\Theta_{\mathrm{FOV}},\Theta_{\mathrm{FOV}}\right]\setminus \underset{k}{⋃}\left[\alpha_{k}^{L},\alpha_{k}^{R}\right]$$

where $\alpha_{k}^{L}$ and $\alpha_{k}^{R}$ denote the left and right angular boundaries of the blocked interval induced by obstacle *k* in the local angular frame centered at $\psi_{i}$.

Thus, local obstacle avoidance is converted into selecting a feasible deviation angle inside the free gaps, following the general idea of gap-based obstacle avoidance [31]. We distinguish interior gaps from boundary gaps adjacent to $\pm \Theta_{\mathrm{FOV}}$.

Within each remaining gap, a small set of candidate deviation angles is sampled, optionally including α=0 when the reference direction lies inside the gap. Candidates are discarded if they are too close to the field-of-view boundary, provide insufficient forward progress, or fail to maintain enough clearance. For each feasible candidate α, we evaluate its normalized clearance ${\tilde{d}}_{i}\left(\alpha \right)$, the normalized width ${\tilde{w}}_{i}\left(\alpha \right)$ of its parent gap, the forward progress term $p_{i}\left(\alpha \right)=\cos\alpha$, and the normalized turning cost $t_{i}\left(\alpha \right)=\left|\alpha \right|/\Theta_{\mathrm{FOV}}$. A binary indicator $b_{i}\left(\alpha \right)\in \left\{0,1\right\}$ marks whether the candidate belongs to a boundary gap.

The local avoidance direction is selected by the lightweight feasible gap score:

(9) $$S_{i}\left(\alpha \right)=k_{\mathrm{clear}}{\tilde{d}}_{i}\left(\alpha \right)+k_{\mathrm{width}}{\tilde{w}}_{i}\left(\alpha \right)+k_{\mathrm{prog}}p_{i}\left(\alpha \right)-k_{\mathrm{turn}}t_{i}\left(\alpha \right)-k_{\mathrm{edge}}b_{i}\left(\alpha \right)t_{i}\left(\alpha \right)$$

which favors large clearance, wide gaps, strong forward progress, and modest turning while mildly penalizing boundary gap solutions. The selected deviation angle and obstacle-guided heading are

(10) $$\alpha_{i}^{⋆}=\arg\max_{\alpha }S_{i}\left(\alpha \right),\;\psi_{i}^{o}=\psi_{i}+\alpha_{i}^{⋆}.$$

Finally, the corresponding obstacle-guided planar velocity is

(11) $$v_{i}^{o}=w_{i2}∥v_{e}∥\left[\begin{array}{c} \cos\psi_{i}^{o}\\ \sin\psi_{i}^{o} \end{array}\right].$$

## 3. Multi-Objective Optimization Formulation and Online Modified MPIO Solver

At each control step, we optimize neither a full trajectory nor low-level actuation directly; instead, UAV *i* searches for a two-dimensional objective-weight vector $w_{i}\in {\left[0,1\right]}^{2}$ that balances the flocking and obstacle avoidance components. In the modified MPIO solver, each pigeon represents one such candidate weight vector. For a given candidate, the controller computes the corresponding blended flocking–avoidance action and the resulting next-step planar state under the current local state. The objective functions introduced below are then used to evaluate the candidate solution represented by that pigeon, i.e., to assess how suitable the induced next-step state is for the controller update of UAV *i*. Therefore, this section formulates the resulting constrained per-step multi-objective problem and describes the modified MPIO teacher used to solve it online, then shows how the selected weight generates the control command and updates the UAV state.

Specifically, for UAV *i*, we define the two-dimensional weight vector

(12) $$w_{i}={\left[w_{i1},\;w_{i2}\right]}^{⊤}\in {\left[0,1\right]}^{2}.$$

### 3.1. Objective Optimization and Feasible Pareto Selection

The first soft objective smoothly shifts from cruise-velocity matching far from obstacles to preservation of forward progress near obstacles:

(13) $$j_{1,i}\left(w_{i}\right)=\beta_{i}\;\left(∥v_{e}∥-v_{i}^{⊤}\frac{v_{e}}{∥v_{e}∥}\right)+\left(1-\beta_{i}\right)∥v_{e}-v_{i}∥$$

where $\beta_{i}\in \left[0,1\right]$ is a clipped coefficient for clearance scheduling determined by the current minimum clearance to the inflated obstacle boundary, with the inflation defined by the hard safety margin $R_{\lim}^{2}$. As the clearance decreases, $\beta_{i}$ increases towards 1, whereas in open space it decreases towards 0.

The second soft objective measures flocking quality and local velocity agreement over the communicated neighborhood. This objective combines the quality of formation geometry and the alignment with neighbor velocity, as follows:

(14) $$\begin{array}{cc} j_{2,i}\left(w_{i}\right)\; & =\sum_{\begin{array}{c} j\in N_{i}\\ ∥p_{j}-p_{i}∥\leq R_{\mathrm{comm}} \end{array}}\left(∥R_{\mathrm{des}}-d_{ij}∥+∥v_{i}-v_{j}∥\right). \end{array}$$

The hard constraints are used as a feasibility screen. The hard constraint for obstacles checks whether the distance between the UAV and the obstacle center is smaller than the obstacle safety radius:

(15) $$j_{3,i}\left(w_{i}\right)=\left\{\begin{array}{cc} 1, & \exists \;k\in K_{\mathrm{obs}}\;\mathrm{such}\;\mathrm{that}\;∥p_{i}-o_{k}∥\leq R_{\lim,k}^{2},\\ 0, & \mathrm{otherwise}. \end{array}\right.$$

The inter-UAV hard constraint checks whether the minimum distance to any neighbor is smaller than the inter-UAV safety radius:

(16) $$j_{4,i}\left(w_{i}\right)=\left\{\begin{array}{cc} 1, & \min_{j\in N_{i}}∥p_{j}-p_{i}∥<R_{\lim}^{1},\\ 0, & \mathrm{otherwise}. \end{array}\right.$$

Only candidates that satisfy both hard constraints are retained for further comparison.

The resulting decision is treated as a constrained bi-objective problem in the Pareto sense [12,13]. Therefore, the per-step decision problem for a single UAV can be written as

(17) $$\min\;\left(j_{1,i}\left(w_{i}\right),\;j_{2,i}\left(w_{i}\right)\right)\;s.t.\;j_{3,i}\left(w_{i}\right)=0,\;j_{4,i}\left(w_{i}\right)=0.$$

### 3.2. Online Modified MPIO Teacher

The modified MPIO solver acts as an online teacher over the weight space. At each step, it evaluates candidate weights, removes infeasible ones using the two hard constraints, performs Pareto ranking on the feasible set with respect to $\left(j_{1,i},j_{2,i}\right)$, and updates the population through leader–follower refinement. The final output is the feasible weight minimizing $j_{2,i}$ on the last Pareto front; if this front is empty, the solver falls back to the best feasible weight in the elite archive *A*, and otherwise to a conservative default weight. Algorithm 1 summarizes the teacher-side per-step weight-selection procedure, and Table 1 lists the corresponding modified-MPIO hyperparameters.

Let $F_{1}^{\left(k\right)}$ denote the Pareto first front at iteration *k*. The landmark center is defined as

(18) $$X_{\mathrm{center}}^{\left(k\right)}=\frac{1}{|F_{1}^{\left(k\right)}|}\sum_{X\in F_{1}^{\left(k\right)}}X,$$

where X denotes a candidate position in the weight space.

A representative form of the implemented leader update combines map-and-compass decay with attraction to an elite representative and to the current landmark center:

(19) $$\begin{array}{c} V_{i}^{\left(k\right)}=e^{-Rk}V_{i}^{\left(k-1\right)}+r_{1}f_{t}\left(1-\frac{\log k}{\log K_{\max}}\right)\left(X_{g}^{\left(k-1\right)}-X_{i}^{\left(k-1\right)}\right)\\ \;+r_{2}f_{t}\left(\frac{\log k}{\log K_{\max}}\right)\left(X_{\mathrm{center}}^{\left(k-1\right)}-X_{i}^{\left(k-1\right)}\right), \end{array}$$

(20) $$X_{i}^{\left(k\right)}=\Pi_{\left[X_{L},X_{U}\right]}\;\left(X_{i}^{\left(k-1\right)}+\Pi_{\left[V_{L},V_{U}\right]}\left(V_{i}^{\left(k\right)}\right)\right),$$

where $X_{g}$ is the current elite representative, *R* is the map-and-compass decay factor, $f_{t}$ is the transition factor, and Π denotes the boundary projection operator. The random coefficients $r_{1}$ and $r_{2}$ are independently drawn from U(0,1).

For ordinary followers, the “modified” component lies in the hierarchical learning rule: lower-ranked individuals do not directly follow the global best, but instead learn a randomly selected dimension from a better-ranked individual. A representative update form is

(21) $$X_{i}^{\left(k\right)}\left(d^{*}\right)=X_{j}^{\left(k-1\right)}\left(d^{*}\right)+e\;r,\;N_{j}^{o}<N_{i}^{o}.$$

In this expression, $d^{*}$ is the randomly selected dimension, *j* is a learning target ranked above *i*, *e* is the learning error, and r∼U(−1,1) is a scalar random perturbation. This mechanism improves information transfer within the population while preserving high-quality Pareto structure and preventing all individuals from collapsing too quickly toward the same local preference.
**Algorithm 1** Online Modified MPIO Teacher for Per-step Weight Selection**Require:** 
Local state of UAV *i*, neighbor states, params $P,K_{d},K_{\max}$**Ensure:** 
Teacher weight $w_{i}^{⋆}$  1:Initialize pop. $P={\left\{X_{q},V_{q}\right\}}_{q=1}^{P}$ in ${\left[0,1\right]}^{2}$ and inject previous valid weight.  2:Evaluate costs for all pigeons. Mark infeasible candidates ($j_{3,i}=1$ or $j_{4,i}=1$) to exclude them from Pareto ranking; init elite archive A←∅.  3:**for** $k=1,\ldots ,K_{\max}$ **do**  4:    Pareto-sort the feasible subset of P, compute landmark center $X_{\mathrm{center}}^{\left(k\right)}$ via Equation (18).  5:    Update $A\leftarrow \mathrm{ArchiveUpdate}(A,F_{1}^{\left(k\right)})$ and select global guidance $X_{g}^{\left(k\right)}$ from $F_{1}^{\left(k\right)}$.  6:    **for** each pigeon q∈P **do**  7:        Store $X_{q}^{\mathrm{old}}$.  8:        **Update:** If *q* is a leader, update via Equations (19) and (20); else perform hierarchical follower update (Equation (21)) or random walk.  9:        **Evaluate & Rollback:** Project to bounds and evaluate costs. Mark if infeasible. If dominated by its previous state, roll back to $X_{q}^{\mathrm{old}}$ and restore costs.10:    **end for**11:    Remove the worst $K_{d}$ pigeons based on Pareto rank and crowding distance to reduce |P|.12:**end for**13:Extract the final feasible Pareto front set $S_{1}$ from P.14:**return** $w_{i}^{⋆}\in S_{1}$ minimizing $j_{2,i}$, else best feasible in *A*, else default [0.2,0.8].

### 3.3. From Weight Selection to Closed-Loop State Update

The selected weight is not an endpoint of the optimization stage; it is the high-level control decision used at the current step. In the current implementation, this decision is not applied through a direct planar Euler update. Instead, the controller retains a quadrotor-compatible control-state separation. The planar input is then constructed as

(22) $${\bar{u}}_{xy,i}=\left[\begin{array}{c} {\bar{u}}_{x,i}\\ {\bar{u}}_{y,i} \end{array}\right]=\left[\begin{array}{c} {\dot{v}}_{i1}^{\mathrm{flock}}+\left(v_{i1}^{o}-v_{i1}\right)\\ {\dot{v}}_{i2}^{\mathrm{flock}}+\left(v_{i2}^{o}-v_{i2}\right) \end{array}\right],$$

and the corresponding autopilot-style planar intermediate references are

(23) $$\left\{\begin{array}{cc} {\bar{v}}_{xy,i} & =∥v_{xy,i}∥+t_{v}\;\left({\bar{u}}_{x,i}\cos\psi_{i}+{\bar{u}}_{y,i}\sin\psi_{i}\right),\\ {\bar{\psi }}_{i} & =\psi_{i}+\frac{t_{\psi }}{{\bar{v}}_{i}}\;\left(-{\bar{u}}_{x,i}\sin\psi_{i}+{\bar{u}}_{y,i}\cos\psi_{i}\right). \end{array}\right.$$

The vehicle state is then propagated over one control period by the corresponding channel dynamics, followed by discrete state advancement of $\left(x_{i},y_{i},v_{xy,i},\psi_{i}\right)$ over the current control period. Teacher-side candidate evaluation uses this mapping to predict the one-step consequence of each candidate weight, whereas the executed closed-loop controller uses the same high-level command semantics to advance the actual system state after a final weight has been selected.

Accordingly, modified MPIO is used to search the control weight vector online for the per-step multi-objective problem. However, because this search is performed under a fixed budget, the feasible front may become sparse under dense interactions or in narrow feasible regions. This motivates replacing the online search with a neural surrogate, while keeping the control execution and state update unchanged.

## 4. Neural Surrogate Learning

**Figure 3:**
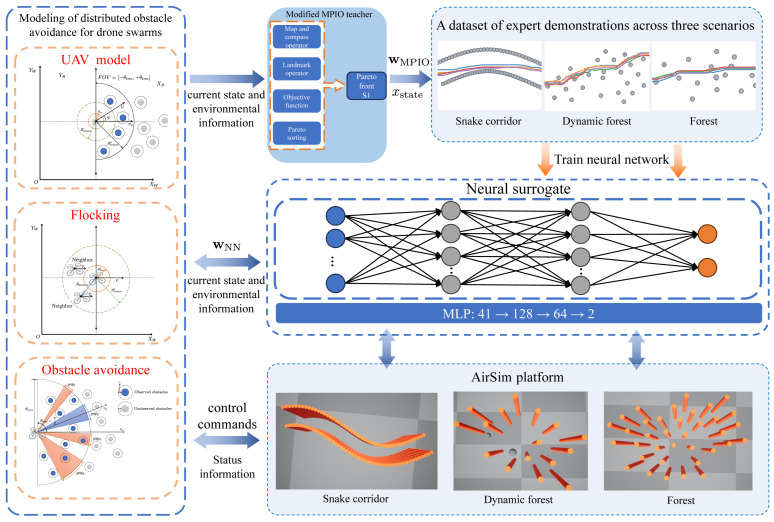
Teacher–student learning framework for surrogate training.

**Figure 4:**
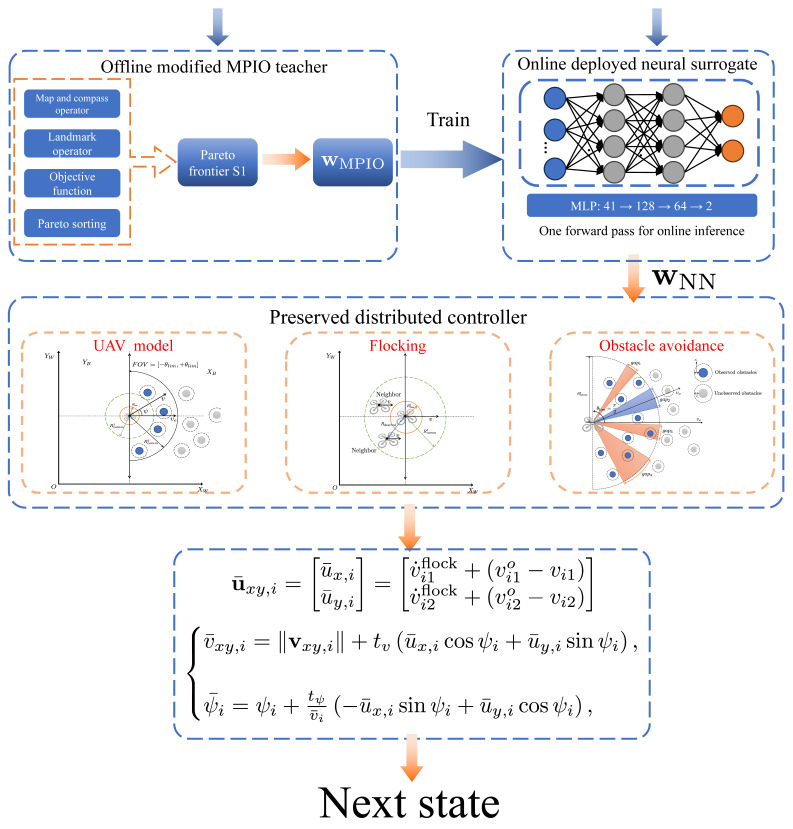
Online control flow of the proposed surrogate-based controller.

To replace the online weight search, we train a neural surrogate that maps local feature vectors to the corresponding weight decisions produced by the online modified MPIO controller. The training set combines base rollouts generated by this controller with DAgger-style relabeling on states visited by the current student. The overall pipeline is illustrated in Figure 3 and the corresponding online control flow used during deployment is summarized in Figure 4.

### 4.1. Base Data Collection and DAgger Relabeling

The training data are constructed by rolling out the online modified MPIO controller in randomized scenes and recording local feature–weight pairs [32]. In this context, the online modified MPIO controller is treated as the teacher, while the neural surrogate is treated as the student. Each sample consists of a 41 dimensional local feature vector and the corresponding two-dimensional weight label. However, successful teacher rollouts under-represent crowded and safe states, especially in narrow passages and densely coupled local interactions. Therefore, we further adopt a DAgger-style data aggregation strategy [26] in which the current student is rolled out closed-loop in randomized scenes and the visited states are converted into local features before being relabeled by the teacher, producing additional feature–weight pairs. The final training set is the union of the base data and the relabeled data.

Formally, let the base imitation dataset be

(24) $$D_{\mathrm{base}}={\left\{\left(x_{n},w_{n}^{⋆}\right)\right\}}_{n=1}^{N_{\mathrm{base}}},\;w_{n}^{⋆}=\pi_{T}\left(x_{n}\right),$$

where $x_{n}\in R^{41}$ denotes the local feature vector defined in Table 2 and $w_{n}^{⋆}\in {\left[0,1\right]}^{2}$ denotes the two-dimensional weight label provided by the online modified MPIO teacher. Let the teacher-relabeled data induced by the current student rollout be

(25) $$D_{\mathrm{dag}}={\left\{\left(x_{m}^{†},w_{m}^{†}\right)\right\}}_{m=1}^{N_{\mathrm{dag}}},\;w_{m}^{†}=\pi_{T}\left(x_{m}^{†}\right),$$

where $x_{m}^{†}$ denotes a state actually visited by the student in closed-loop and then relabeled by the teacher. The final mixed dataset is defined as

(26) $$D_{\mathrm{mix}}=D_{\mathrm{base}}\cup D_{\mathrm{dag}}.$$

Following this procedure, the retained mixed-dataset artifact used for training adds 70 DAgger-style episodes on top of the 70 retained base episodes, yielding 140 episodes and 53,747 supervised samples.

### 4.2. Surrogate Network and Risk-Weighted Training Objective

We use a lightweight multilayer perceptron as the neural surrogate to directly learn the mapping from local state features to the teacher weight vector. Let the input feature be x. Before training, each feature dimension is standardized as

(27) $$\tilde{x}=\frac{x-\mu }{\sigma },$$

where μ and σ respectively denote the feature-wise mean and standard deviation estimated from the training set. The surrogate policy is denoted by

(28) $$\hat{w}=f_{\theta }\left(\tilde{x}\right)$$

and implemented as the lightweight MLP

(29) 41→128→64→2.

Each training sample pairs a standardized 41-dimensional local feature vector with the corresponding teacher-selected objective-weight vector. The surrogate is implemented as an MLP with ReLU activations in the hidden layers and a two-neuron sigmoid output layer, which constrains the predicted weights to ${\left[0,1\right]}^{2}$. The network output is not a velocity, acceleration, or low-level UAV command; instead, it replaces the online modified MPIO weight search result and specifies the relative emphasis between the preserved flocking/formation component and the preserved gap-based obstacle avoidance component. The resulting weights are then used by the unchanged downstream command generation and state update rules. Neighbor and obstacle features are constructed in an ego-centered local frame before being fed to the MLP. Neighbor entries are ordered by increasing Euclidean distance to the ego UAV, and obstacle entries are ordered by increasing distance from the ego UAV to the obstacle center. The input keeps only the nearest three neighbors and nearest three obstacles; if fewer than three entries are available, zero padding is used to keep the 41-dimensional input fixed. Apart from this top-*K* selection and padding, the raw geometric, velocity, radius, and clearance features are not manually clipped, min–max normalized, or hard-bounded before training. Instead, each input dimension is standardized using the training-set mean and standard deviation, as provided in Equation (27). The sigmoid bound is applied only to the two-dimensional output weight vector, not to the input features. Auxiliary vertical quantities such as $\lambda_{i}$ and $h_{i}$ are retained only to provide context for altitude regulation towards $h_{e}$ and the current operating condition; the surrogate prediction itself remains associated with the planar high-level weight-selection decision.

Training uses a risk-weighted SmoothL1 loss

(30) $$L=\frac{1}{B}\sum_{b=1}^{B}\omega_{b}\;\ell_{\mathrm{SmoothL}1}\left({\hat{w}}_{b},w_{b}^{⋆}\right),$$

where *B* is the mini-batch size, $w_{b}^{⋆}$ is the teacher weight label, and ${\hat{w}}_{b}$ is the surrogate output. The factor $\omega_{b}$ is a per-sample training weight used to emphasize safety-critical states. It is increased for samples with small neighbor or obstacle safety margins, and is further amplified for samples drawn from episodes containing inter-UAV or obstacle collisions.

Model selection uses validation loss with early stopping. The offline test MAE is 0.1929 and is reported only as an indicator of teacher-weight fitting quality, whereas the main evidence remains closed-loop safety and real-time performance.

## 5. Results and Discussion

### 5.1. Experimental Protocol

All controllers are evaluated on the same retained scene instances using the same simulator step size (Δt = 0.5 s), episode horizon of 59.5 s, and metric definitions. The closed-loop benchmark uses synchronous simulation. At each control step, the simulator waits for the controller output before advancing to the next step. The retained mixed dataset used for training contains 140 episodes, which are split at the episode level into 80% training, 10% validation, and 10% test subsets. The base dataset and DAgger dataset use random seeds 2027 and 3010, respectively, whereas the benchmark evaluation uses seeds 2028, 2029, and 2030. This retained benchmark contains 450 closed-loop episodes in total, and is used as a controlled and reproducible evaluation set for method comparison rather than as a comprehensive robustness study over broad random-seed coverage.

All simulation, dataset generation, and closed-loop evaluation code was implemented in Python 3.9. The experiments were conducted on a local laptop equipped with a 13th Gen Intel Core i7-13700H CPU and 16 GB RAM. Neural network training used an NVIDIA GeForce RTX 4060 Laptop GPU with 8 GB VRAM. Closed-loop latency profiling was performed under the same hardware setting.

The reported metrics are true collision-free rate, safe success rate, formation pass rate, whole-swarm step compute time, and step–overrun ratio. The true collision-free rate requires that no inter-UAV hard boundary violation and no obstacle hard boundary violation occur at any step. The safe success rate is stricter; in addition to true collision-free execution, it also requires no violation of the inflated obstacle boundary with a margin of 0.5 m. The formation pass rate counts the episodes for which the closed-loop formation error remains within the same prescribed benchmark thresholds throughout the episode; the mean formation error must not exceed 3.0 m, the maximum formation error must not exceed 10.0 m, and the fraction of over-limit steps must not exceed 0.20. The whole-swarm step compute time is defined as the sum of all local decision times within one simulation step. Finally, the step–overrun ratio is defined consistently with this whole-swarm latency, i.e., as the fraction of simulation steps for which the whole-swarm decision time exceeds the control step size Δt = 0.5 s. Therefore, the reported latency is used only as a profiling metric for the current implementation, and does not alter the benchmark state update.

Table 3 summarizes the closed-loop control settings and gap-selection coefficients used in the benchmark, including the task ranges, vehicle dynamics, swarm-interaction parameters, safety radii, sensing range, and gap-selection weights. Table 4 lists the surrogate training, hardware, and risk-weighting settings, including the optimizer hyperparameters, computing platform, and per-sample training-weight coefficients.

### 5.2. Closed-Loop Simulation Results

Overall Quantitative Results.

Table 5 summarizes the overall closed-loop comparison. Under the synchronous closed-loop benchmark, the neural surrogate outperforms both modified MPIO and base MPIO on the primary safety metrics while reducing both whole-swarm step compute time and step–overrun ratio by a large margin. Relative to modified MPIO, the neural surrogate improves the true collision-free rate from 48.00% to 86.89%, raises the safe success rate from 38.67% to 74.22%, and increases the formation pass rate from 30.44% to 34.44%. At the same time, the mean whole-swarm step compute time decreases from 8494.70 ms to 0.92 ms, and the step–overrun ratio drops from 98.75% to 0.00%. The base-MPIO baseline is weaker still across the same overall comparison, with a 98.93% step–overrun ratio. Taken together, these results on the retained frozen benchmark used in this study show that replacing the online modified MPIO weight search with the neural surrogate mainly improves the reported safety and real-time metrics, especially collision avoidance and step-level computation time, while the formation pass gain is comparatively modest. Under the current implementation, this also makes the controller compatible with the Δt = 0.5 s step budget.

Additional Seed Sensitivity Check.

To further examine seed sensitivity, we evaluated the final neural surrogate on three additional evaluation seeds not included in the retained benchmark: 1000, 2000, and 3000. We used the same three scene families and 50 episodes per scene, resulting in 450 additional closed-loop episodes. As shown in Table 6, the additional seeds produced a true collision-free rate of 87.78±0.38%, a safe success rate of 74.67±4.16%, and a formation pass rate of 34.00±1.15%, which are close to the retained benchmark results over seeds 2028, 2029, and 2030. These results suggest that the final surrogate behavior is not limited to the original three evaluation seeds. Nevertheless, this additional check is reported as a seed sensitivity analysis rather than as a comprehensive robustness validation over broad random seed coverage.

Cross-Scene Consistency.

**Figure 5:**
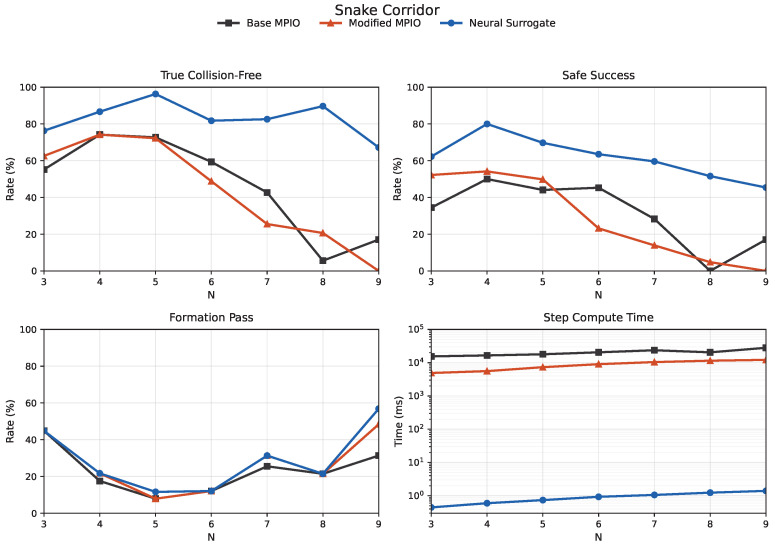
Bucketed closed-loop metrics in the snake corridor scene.

**Figure 6:**
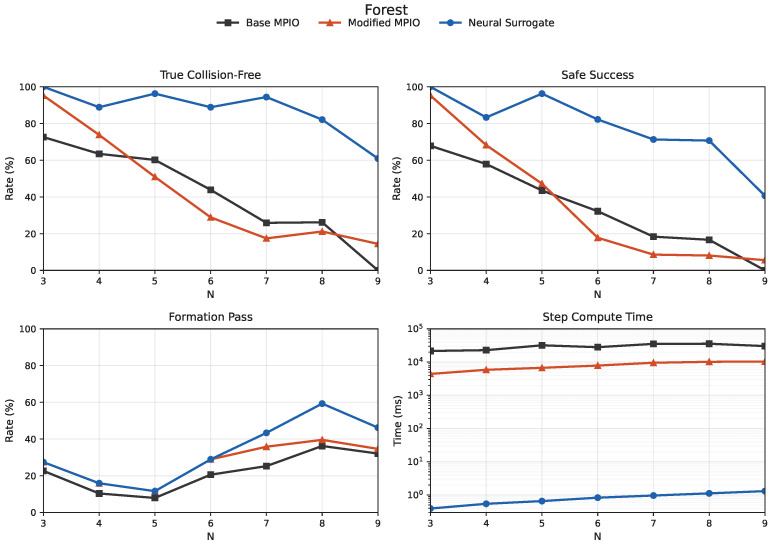
Bucketed closed-loop metrics in the forest scene.

**Figure 7:**
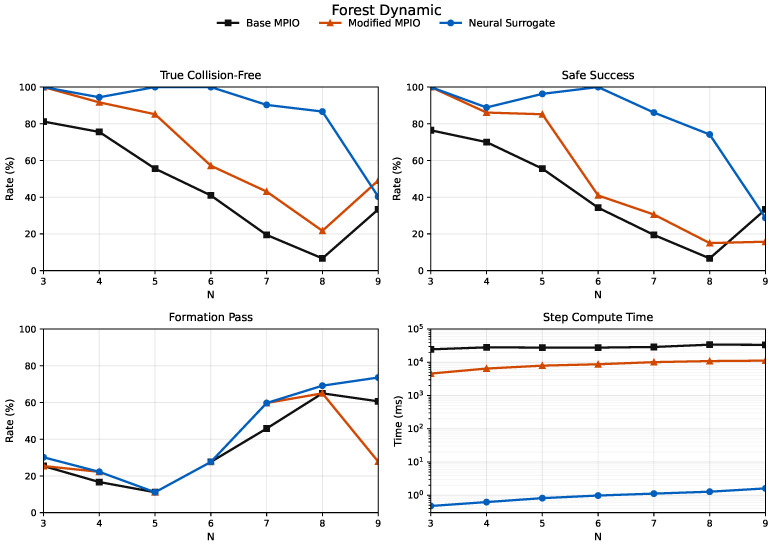
Bucketed closed-loop metrics in the forest and dynamic obstacles scene.

The overall advantage is not driven by a single favorable scene family. As shown in Figure 5, Figure 6 and Figure 7, the neural surrogate generally maintains stronger safety performance across the benchmark scene families, particularly in true collision-free rate and safe success rate, while its step compute time remains separated from both optimization-based baselines by several orders of magnitude. The formation pass rate does not show the same level of improvement as the safety and latency metrics, but remains broadly comparable across the tested scene families. Thus, the surrogate not only appears better after averaging over all cases, its main advantages in safety and online computation also remain visible as the environment becomes more cluttered and as the swarm size increases from N=3 to N=9.

Failure Mode Breakdown.

To clarify where the collision-free improvement comes from, Table 7 decomposes the hard-collision statistics into inter-UAV collisions and obstacle hard collisions. The main difference appears on the inter-UAV side. The modified MPIO shows a 44.00% neighbor collision rate but only an 8.00% obstacle hard-collision rate; by contrast, the neural surrogate reduces the neighbor collision rate to 5.56% while keeping a comparable obstacle hard-collision rate of 7.56%. Therefore, the increase in true collision-free rate from 48.00% to 86.89% is driven primarily by improved inter-UAV safety rather than by a large change in obstacle hard-collision performance. This table explains the main source of the true collision-free improvement. The remaining gap between true collision-free and safe success is governed by the stricter inflated boundary margin criterion defined in Section 5.1, which is not decomposed in Table 7.

One notable observation in Table 5 is that the neural surrogate outperforms its online teacher even under the synchronous benchmark. This does not mean that a learned student generally outperforms an exact expert, because the comparison here is not against an exact per-step optimum but against a finite-budget online approximate teacher. In the current implementation, the teacher must complete feasibility screening and Pareto search within the fixed budget $K_{\max}=20$, so difficult local states can lead to sparse feasible fronts and unstable final selections. Therefore, we interpret the student-over-teacher gap as an empirical consequence of replacing a budget-limited online solver with a smoother learned approximation on the tested closed-loop state distribution.

Training and Model Ablations.

Table 8 reports ablations on the training protocol and surrogate capacity under the same retained frozen benchmark of 450 episodes. Scene randomization is kept fixed across variants because removing it would alter the underlying teacher rollout and dataset generation distribution as opposed to isolating a local training component. The “No DAgger” variant uses only the retained base teacher trajectories, the “Uniform Loss” variant uses the mixed dataset but removes the per-sample risk weights, and the variants with different model sizes keep the same risk-weighted training pipeline while changing only the hidden layer widths.

Together with the preceding comparison against the online modified MPIO teacher, these ablations indicate that the benchmark-level gain mainly comes from replacing the repeated online weight search with a learned surrogate, rather than from any single training refinement alone. Removing DAgger changes the true collision-free rate by only −0.45 percentage points relative to the full model, while replacing the risk-weighted objective with a uniform loss changes it by −0.22 percentage points. These small changes do not support treating either component as the dominant source of the final gain. Risk weighting has a clearer effect on the stricter safe success metric, increasing it from 72.44% to 74.22% relative to uniform loss, even though the full model has a higher offline MAE. This mismatch further supports treating offline teacher-weight fitting error as an auxiliary diagnostic rather than as the main evidence of closed-loop controller quality. The strongest ablation effect comes from surrogate capacity, with both the smaller and larger MLPs reducing collision-free and safe success rates and increasing inter-UAV collision rates relative to the default 128–64 model. Among the tested architectures, the medium-sized surrogate provides the best closed-loop safety tradeoff.

### 5.3. AirSim Case Study

**Figure 8:**
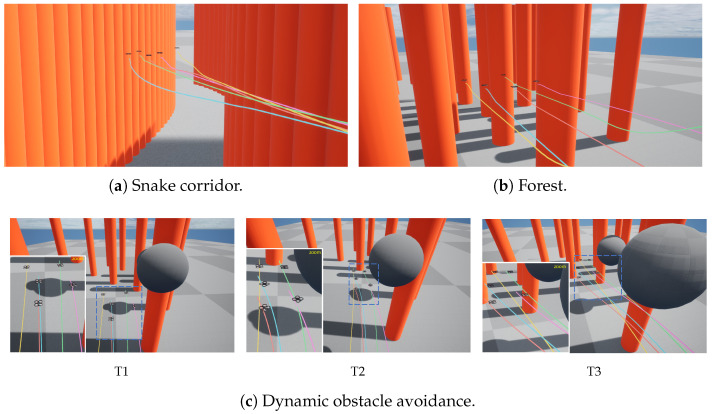
AirSim deployment results for the snake corridor, forest, and dynamic obstacle avoidance scenes. Colored trajectory lines denote different UAVs in the swarm, and the inset shows a zoomed-in view of the local trajectory segment.

Figure 8 presents an AirSim case study of the neural surrogate controller. This case study examines whether the surrogate-based high-level controller remains executable after migration from the point-mass benchmark to a higher-fidelity asynchronous multirotor simulator. As such, it serves as qualitative evidence of the migration feasibility and real-time plausibility of the learned controller.

**Figure 9:**
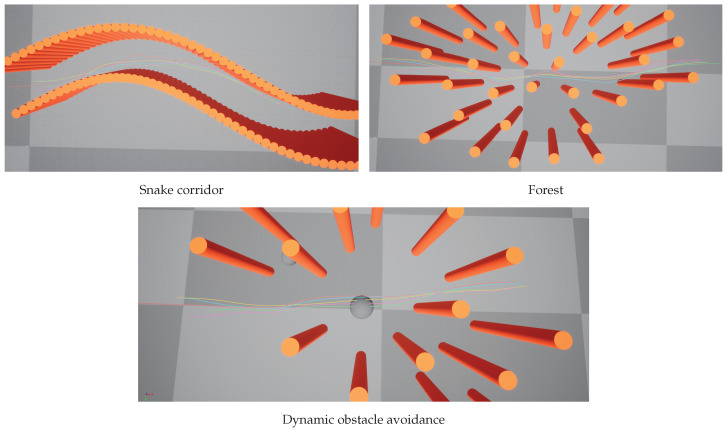
Top-view trajectories of the deployed neural surrogate controller in the three representative AirSim scenes. Colored trajectory lines denote different UAVs in the swarm.

The neural surrogate controller remains executable in AirSim and maintains stable closed-loop behavior in representative cluttered scenes. Figure 9 further shows the complete top-view trajectories in the three representative AirSim scenes, providing a clearer view of the overall path shapes, obstacle circumvention patterns, and trajectory continuity of the deployed controller. Together, these qualitative results show that the surrogate is not only effective on the frozen point-mass benchmark but is also suitable for deployment-oriented execution in a simulator with asynchronous updates and multirotor command interfaces. This evidence is qualitative, whereas the main quantitative comparison among controllers is provided by the frozen synchronous benchmark in Section 5.2.

We do not use the AirSim case study as a quantitative baseline comparison against base MPIO or modified MPIO. Under the current integration, the online optimization baselines require substantially longer per-step decision times than the neural surrogate, and would need additional simplifications or reduced search settings to run stably through the same asynchronous multirotor interface. Therefore, the fair controller comparison is kept in the retained synchronous benchmark, where all methods use the same scene instances and metric definitions. The AirSim figures are reported only to show that the learned high-level surrogate controller can be executed through a higher-fidelity multirotor simulation interface.

## 6. Conclusions

This paper develops a neural surrogate replacement for the online weight selection component within an inherited modified MPIO-based swarm controller rather than a new low-level quadrotor controller or a full end-to-end swarm-control architecture. The learned surrogate preserves the surrounding high-level control structure; this allows it to avoid repeated online optimization during deployment, which can make the teacher difficult to deploy under strict runtime budgets. On the closed-loop benchmark, the neural surrogate improves the true collision-free rate and safe success rate relative to both modified MPIO and base MPIO, modestly improves the formation pass rate, and reduces the whole-swarm step compute time and step–overrun ratio by a large margin. A breakdown of the safety results shows that the gain in true collision-free rate is driven mainly by better inter-UAV safety. The ablation results further indicate that the benchmark-level improvement is not dominated by any single training refinement alone; instead, the clearest effect comes from replacing the finite-budget online modified MPIO search with a learned medium-sized surrogate, while DAgger and risk-weighted supervision mainly support state coverage and safety emphasis during training. A qualitative AirSim case study further suggests that the learned high-level controller remains executable after migration to a higher-fidelity asynchronous multirotor simulator. A remaining limitation is that the present manuscript does not provide a directly comparable real-time deployment baseline for modified MPIO under the same interface as the neural surrogate. Our preliminary AirSim attempts indicate that stable execution of modified MPIO would require substantially reduced search settings and simplified scenarios under the current integration. In addition, the learned controller has not yet been validated on real hardware. The present results should be interpreted as evidence on a retained closed-loop benchmark rather than as a comprehensive robustness validation across broad random-seed coverage. Extending the evaluation along these directions is an important next step for strengthening both the deployment conclusions and the mechanism-level interpretation of this work.

## Figures and Tables

**Table 1:** Online modified MPIO teacher hyperparameters.

Symbol	Value	Notes
*P*	58	Population size
$K_{\max}$	20	Max iterations per step
$K_{d}$	2	Removed per iteration
$A_{\max}$	50	Elite archive size
*R*	0.3	Leader decay factor
$f_{t}$	3.0	Exploration-convergence factor
$p_{l}$	0.9	General-leader ratio
$e_{\mathrm{learn}}$	0.01	Follower-learning perturbation
$s_{l}$	2	Follower-learning repetitions
$e_{\mathrm{rand}}$	0.01	Fallback random-walk amplitude
$X_{L}$	${\left[0,0\right]}^{⊤}$	Lower bound of the 2-D weight position
$X_{U}$	${\left[1,1\right]}^{⊤}$	Upper bound of the 2-D weight position
$V_{L}$	${\left[-0.2,-0.2\right]}^{⊤}$	Lower bound of the 2-D pigeon velocity
$V_{U}$	${\left[0.2,0.2\right]}^{⊤}$	Upper bound of the 2-D pigeon velocity
Δt	0.5 s	Step size
$T_{\mathrm{epi}}$	59.5 s	Episode horizon

**Table 2:** Definition of the 41-dimensional surrogate input vector.

Indices	Dim.	Content
1–6	6	Ego planar velocity $\left(v_{x,i},\;v_{y,i}\right)$, speed $∥v_{i}∥$, heading $\psi_{i}$, and vertical-state information given by altitude rate $\lambda_{i}$ and altitude $h_{i}$.
7–8	2	Desired cruise velocity $\left(v_{e,x},\;v_{e,y}\right)$
9–23	3×5	Up to three nearest neighbors: relative position, relative velocity, and distance $\left(\Delta x_{ij},\;\Delta y_{ij},\;\Delta v_{x,ij},\;\Delta v_{y,ij},\;d_{ij}\right)$, with $d_{ij}=\sqrt{\Delta x_{ij}^{2}+\Delta y_{ij}^{2}}$
24–41	3×6	Up to three nearest obstacles: relative center position, radius, planar velocity, and clearance $\left(\Delta x_{io},\;\Delta y_{io},\;r_{o},\;v_{o,x},\;v_{o,y},\;c_{io}\right)$, with $c_{io}=d_{io}-r_{o}$

**Table 3:** Closed-loop control settings and gap selection coefficients. Ranges denote episode-level perturbations.

Symbol	Value	Notes
$t_{v}$	0.8	Speed time constant
$t_{\psi }$	0.6	Heading time constant
$v_{xy}$	[4.0,12.0]	Speed range (m/s)
$n_{\max}$	3.0	Max lateral overload (g)
*N*	[3,9]	UAVs per episode
$h_{e}$	[40,70]	Target altitude range (m)
$v_{e}$	$\left(v_{e},0\right),\;v_{e}\in \left[6,10\right]$	Cruise speed range (m/s)
$R_{\mathrm{des}}$	[6,10]	Target spacing range (m)
$R_{\mathrm{comm}}$	40	Neighbor radius (m)
$R_{\lim}^{1}$	2.0	UAV safety radius (m)
$R_{\mathrm{sense}}$	40.0	Obstacle sensing radius (m)
$K_{f}$	0.25	Spacing gain
$K_{a}$	0.1	Velocity-alignment gain
$K_{c}$	$1.0\times 10^{5}$	Inter-UAV collision-repulsion gain
$R_{\lim}^{2}$	10.0	Inflated obstacle safety radius (m)
$\Theta_{\mathrm{FOV}}$	π/2	Gap-selection half FOV
$k_{\mathrm{clear}}$	3.0	Clearance weight in $S_{i}\left(\alpha \right)$
$k_{\mathrm{width}}$	1.0	Gap-width weight in $S_{i}\left(\alpha \right)$
$k_{\mathrm{prog}}$	0.6	Progress weight in $S_{i}\left(\alpha \right)$
$k_{\mathrm{turn}}$	0.2	Turn-cost weight in $S_{i}\left(\alpha \right)$
$k_{\mathrm{edge}}$	0.3	Boundary penalty in $S_{i}\left(\alpha \right)$

**Table 4:** Surrogate training, hardware, and risk weighting settings.

Item	Value	Notes
Batch size	512	Mini-batch size
Learning rate	$10^{-3}$	Initial learning rate
Weight decay	$10^{-4}$	Weight-decay coefficient
Max epochs	60	Max training epochs
Patience	10	Early-stop patience
CPU	13th Gen Intel Core i7-13700H	14 cores, 20 threads, 2.40 GHz
GPU	NVIDIA GeForce RTX 4060 Laptop GPU	8 GB VRAM
$m_{\mathrm{nbr}}^{\mathrm{ref}}$	0.5	Reference neighbor margin in $\omega_{b}$
$m_{\mathrm{obs}}^{\mathrm{ref}}$	1.0	Reference obstacle margin in $\omega_{b}$
$w_{\mathrm{nbr}}$	5.0	Neighbor-margin weight in $\omega_{b}$
$w_{\mathrm{obs}}$	4.0	Obstacle-margin weight in $\omega_{b}$
$c_{\mathrm{nbr}}$	3.0	Neighbor-collision multiplier in $\omega_{b}$
$c_{\mathrm{obs}}$	2.5	Obstacle-collision multiplier in $\omega_{b}$

**Table 5:** Closed-loop comparison on the retained frozen benchmark.

Method	True Collision-Free (%) ↑	Safe Success (%) ↑	Formation Pass (%) ↑	Step Compute Time (ms) ↓	Overrun Ratio (%) ↓
Base MPIO [33]	41.11±10.38	33.11±6.94	26.44±2.52	26,772.83 ± 1396.42	98.93
Modified MPIO [16]	48.00±7.33	38.67±4.37	30.44±2.69	8494.70±273.23	98.75
Ours	86.89±0.77	74.22±2.78	34.44±1.39	0.92±0.10	0.00

*Note:* Upward and downward arrows indicate whether larger or smaller values are preferred, respectively; bold values mark the best result in each metric column.

**Table 6:** Additional seed sensitivity check for the final neural surrogate.

Evaluation Seed	Episodes	True CF (%)	Safe Success (%)	Formation Pass (%)	Neighbor Coll. (%)	Obstacle Hard Coll. (%)	Step Time (ms)	Overrun (%)
2028, 2029, 2030	450	86.89±0.77	74.22±2.78	34.44±1.39	5.56±1.92	7.56±1.68	0.92±0.10	0.00±0.00
1000, 2000, 3000	450	87.78±0.38	74.67±4.16	34.00±1.15	5.56±1.39	6.67±1.33	0.76±0.03	0.00±0.00
1000	150	88.00	78.00	34.67	4.00	8.00	0.74	0.00
2000	150	88.00	70.00	32.67	6.67	5.33	0.80	0.00
3000	150	87.33	76.00	34.67	6.00	6.67	0.76	0.00

**Table 7:** Breakdown of collision types on the retained frozen benchmark.

Method	Neighbor Collision (%) ↓	Obstacle Hard Collision (%) ↓	True Collision-Free (%) ↑
Base MPIO [33]	48.22	10.67	41.11
Modified MPIO [16]	44.00	8.00	48.00
Ours	5.56	7.56	86.89

*Note:* Upward and downward arrows indicate whether larger or smaller values are preferred, respectively; bold values mark the best result in each metric column.

**Table 8:** Training and model ablations on the frozen benchmark.

Variant	Setting	True CF (%)	Safe Success (%)	Formation Pass (%)	Neighbor Coll. (%)	Obstacle Hard Coll. (%)	Step Time (ms)	MAE
Full	DAgger + risk, 128–64	86.89	74.22	34.44	5.56	7.56	0.923	0.1929
No DAgger	Base data + risk, 128–64	86.44	75.33	34.67	6.22	7.33	0.867	0.1996
Uniform loss	DAgger + uniform, 128–64	86.67	72.44	34.67	6.22	7.11	0.846	0.1869
Small MLP	DAgger + risk, 64–32	76.22	64.44	32.00	14.89	8.89	0.809	0.1872
Large MLP	DAgger + risk, 256–128	77.78	69.11	31.33	16.00	6.22	1.678	0.1881

## Data Availability

The datasets generated and analyzed during the current study are not publicly archived. They are available from the first author upon reasonable request. The source code supporting the implementation is available in the public repository cited in the manuscript.
